# Supplementary material for: Prospector Heads: Generalized Feature Attribution for Large Models & Data
Source: ArXiv. 2024 Jun 20:arXiv:2402.11729v2. Preprint. [Version 2] (PMC11213143)
Supplement: Supplement 1 [file NIHPP2402.11729v2-supplement-1.pdf]

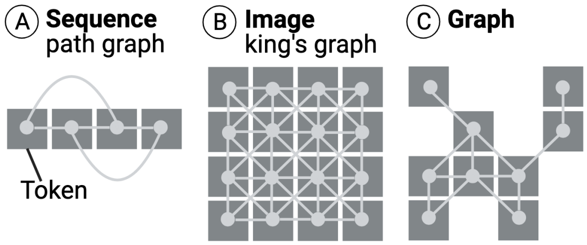

Figure S1: Unstructured data represented as map graphs. Sequences (A) and images (B) require a specified resolution (*e.g.*, words or sentences for text, pixels or patches for images) and connectivity (*e.g.*, 2-hop, 8-way) for discretization.

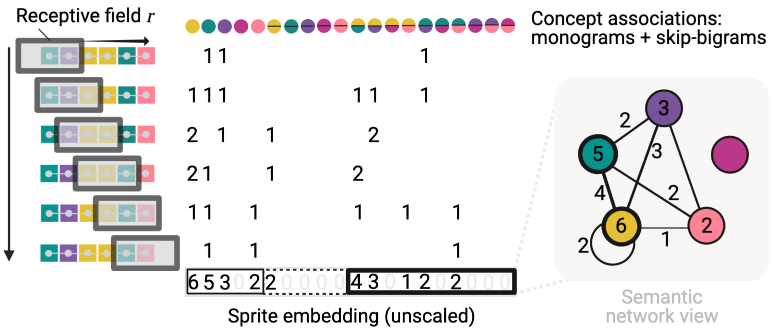

Figure S2: A depiction of the `rollup` operation as seen in layer (II).

Attribution method	Full-context	Partial-context
Gradients	$O(F+B)$	$O(T(F+B))$
CAM (Zhou et al., 2016)	$O(pF)$	$O(TpF)$
GradCAM (Selvaraju et al., 2016)	$O(pF+B)$	$O(T(pF+B))$
ScoreCAM (Wang et al., 2019)	$O(pF)$	$O(TpF)$
Attention	$O(F)$	$O(TF)$
SHAP (Lundberg & Lee, 2017)	$O(F2^T)$	$O(TF2^{T^*})$
DASP (Ancona et al., 2019)	$O(FT^2)$	$O(TFT^{*2})$
G-DeepSHAP (Chen et al., 2022b)	$O(p(F+B))$	$O(Tp(F+B))$
Prospectors (ours)	$O(F+T)$	$O(TF)$

Table S1: Comparison of inference-time computational complexity between attribution methods. Parameters T, T^*, F, B, and p are respectively the number of: tokens, sub-tokens, operations in a forward pass, operations in a backward pass, number of passes.

	CPU time		Wall clock time	
Attribution	token	datum	token	datum
SHAP	25.459	1428.347	19.222	1078.738
Prospectors		0.108		0.108

Table S2: Speed benchmarking between PartitionSHAP (denoted as SHAP) and prospectors, as applied to partial-context encoders and text (WikiSection) data. We report mean values, and SHAP's values reflect a random sample of 86% (621/718) of test-set examples.

Name	WikiSection	Camleyon16	MetalPDB
Token resolution	sentence	224×224 patch	atom
Token connectivity (δ)	2(-hop)	8(-way)	-
Concept count (K)	{10,15,20,25,30}	{10,15,20,25,30}	{15,20,25,30}
Receptive field (r)	{0,1,2,4,8}	{0,1,2,4,8}	{0,1,2,4}
Significance threshold (α)	{0.01,0.025,0.05,∞}	{0.01,0.025,0.05,∞}	{0.001,0.01,0.05,0.5,∞}
Fold-change threshold (τ)	{0,1,2}	{0,1,2}	{0,1,2,4}
Regularization factor (λ)	0.5	0.5	{0.0,0.5,1.0}
Edge cutoff (ϵ)	-	-	{4.0,6.0,8.0}

Table S3: Hyperparameters tuned during training grid search. Note: edge cutoff (ϵ), the distance cutoff to control graph density, only applies to MetalPDB. We use ∞ to represent any large number that acts as a non-threshold.

Encoder Alias	K	r	τ	α	λ	ϵ
MiniLM	25	1	1	0.05	—	—
DeBERTa	—	—	—	—	—	—
tile2vec	20	8	0	∞	—	—
ViT	20	2	0	0.05	—	—
CLIP	30	2	2	∞	—	—
PLIP	15	1	2	0.01	—	—
COLLAPSE	25	4	4	1.0	—	8.0
ESM2	30	1	0	1.0	—	8.0
AA	21*	1	1	0.1	1.0	6.0

Table S4: Top prospectors per encoder, after model selection and sequential ranking. All selected prospectors except AA are parameter-free fold-change variants. *The AA encoder does not use clustering for quantization, since amino acids are already a discrete set of 21 tokens (20 standard amino acids + 1 entry for any non-standard amino acid). Symbol "—" denotes non-applicable hyperparameter.

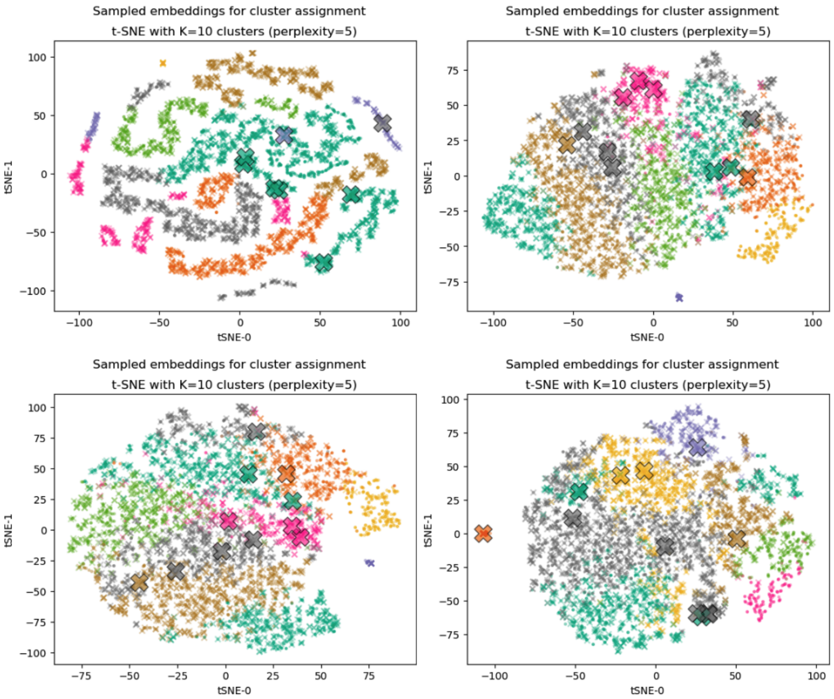

Figure S3: t-SNE plots, left-to-right then top-to-bottom: tile2vec, ViT, CLIP, PLIP. Marker color denotes concept, marker type denotes ground truth annotation for a patch: $\circ$ for class_0, $\times$ for patches from class_1 images that do originate from target regions, and the much thicker $\boldsymbol{\times}$ for class_1-specific target regions.

Encoder-Attribution	Mean AUPRC	Error AUPRC	Mean AP	Error AP
DeBERTa-ZSC	0.476	0.032	0.502	0.031
DeBERTa-SHAP	0.292	0.026	0.322	0.026
DeBERTa-NLI	**0.584**	0.030	**0.617**	0.023
DeBERTa-Attention	0.217	0.020	0.244	0.021
MiniLM-SVM	0.284	0.024	0.317	0.024
MiniLM-MLP	*0.626*	0.031	*0.648*	0.030
MiniLM-Prospector	**0.711**	0.030	**0.730**	0.028

Table S5: Tabular results for sequences (WikiSection). Top section contains baseline methods while bottom section contains prospector-equipped encoders. **Boldface** indicates best-in-encoder results. *Italics* indicates top non-prospector pipeline.

Encoder-Attribution	Mean AUPRC	Error AUPRC	Mean AP	Error AP
ViT-Attention	0.158	0.038	0.162	0.038
ViT-Probability	*0.207*	0.043	*0.212*	0.043
CLIP-Probability	0.149	0.035	0.155	0.034
PLIP-Probability	0.163	0.037	0.167	0.037
tile2vec-Prospector	**0.212**	0.044	**0.218**	0.044
ViT-Prospector	**0.210**	0.047	**0.215**	0.047
CLIP-Prospector	**0.330**	0.056	**0.298**	0.055
PLIP-Prospector	**0.470**	0.050	**0.300**	0.052

Table S6: Tabular results for images (Camelyon16). Top section contains baseline methods while bottom section contains prospector-equipped encoders. **Boldface** indicates best-in-encoder results. *Italics* indicates top non-prospector pipeline.

Encoder-Attribution	Mean AUPRC	Error AUPRC	Mean AP	Error AP
COLLAPSE-GNNExplainer	0.242	0.020	*0.266*	0.020
COLLAPSE-Attention	0.171	0.013	0.199	0.013
COLLAPSE-SHAP	0.370	0.015	0.162	0.016
ESM-GNNExplainer	0.036	0.003	0.047	0.004
ESM-Attention	0.050	0.005	**0.064**	0.005
ESM-SHAP	**0.304**	0.015	0.062	0.006
AA-GNNExplainer	0.105	0.015	0.121	0.015
AA-Attention	0.031	0.005	0.043	0.004
AA-SHAP	***0.420***	0.011	0.062	0.008
COLLAPSE-Prospector	**0.640**	0.020	**0.323**	0.039
ESM-Prospector	0.082	0.013	0.060	0.007
AA-Prospector	0.405	0.037	**0.354**	0.037

Table S7: Tabular results for protein graphs (MetalPDB). Top section contains baseline methods while bottom section contains prospector-equipped encoders. **Boldface** indicates best-in-encoder results. *Italics* indicates top non-prospector pipeline.

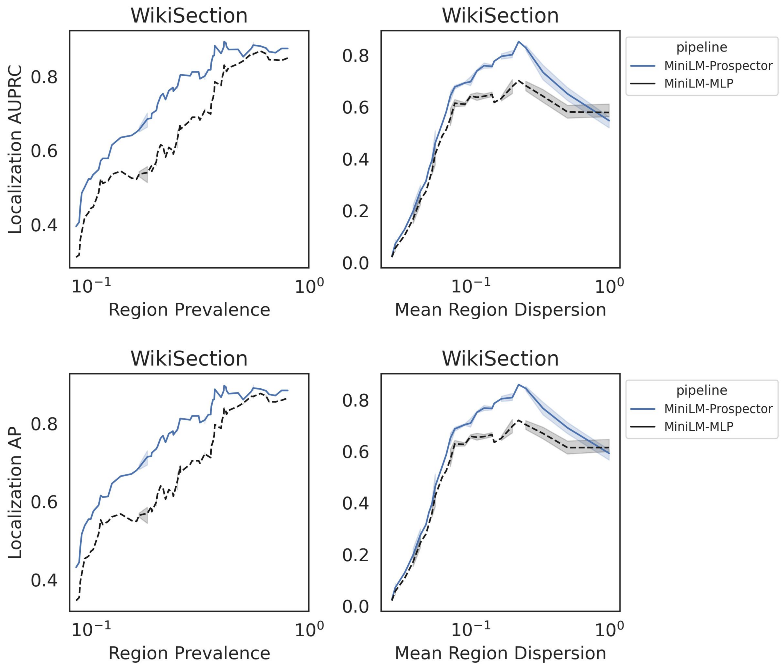

Figure S4: Robustness for WikiSection data. Top baseline, MiniLM encoder with MLP head, is denoted by a black dashed line.

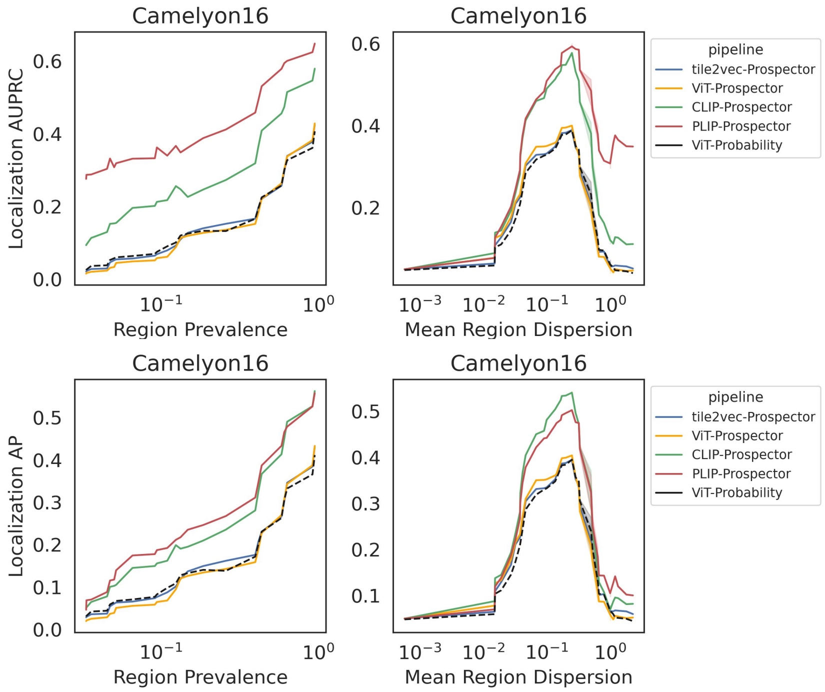

Figure S5: Robustness for Camelyon16 data. Top baseline, ViT encoder with prediction probabilities, is denoted by a black dashed line.

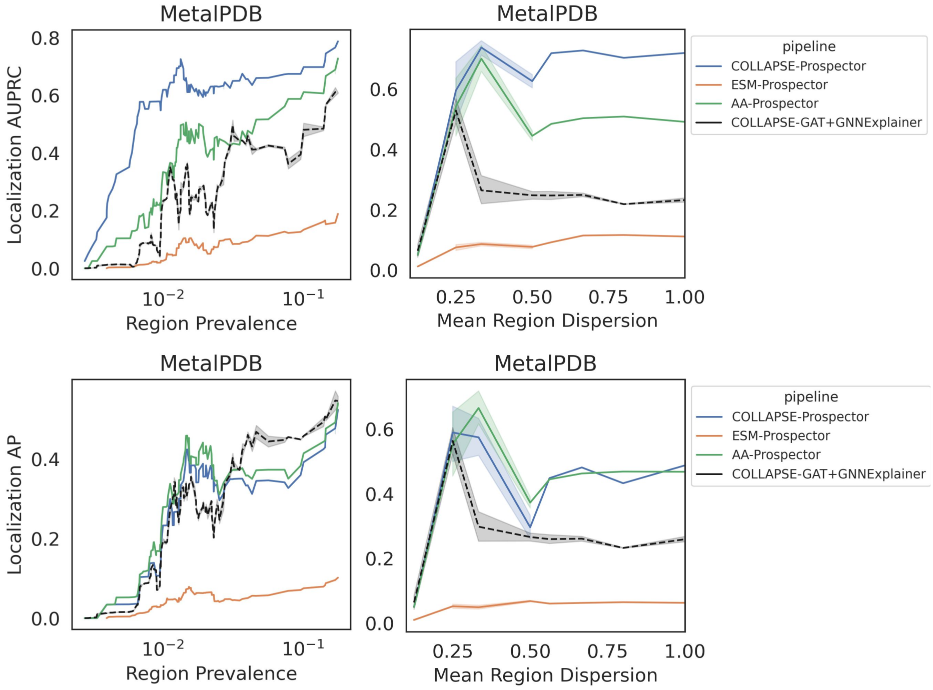

Figure S6: Robustness for MetalPDB data. Top baseline, COLLAPSE encoder with GAT head and GNNExplainer, is denoted by a black dashed line.

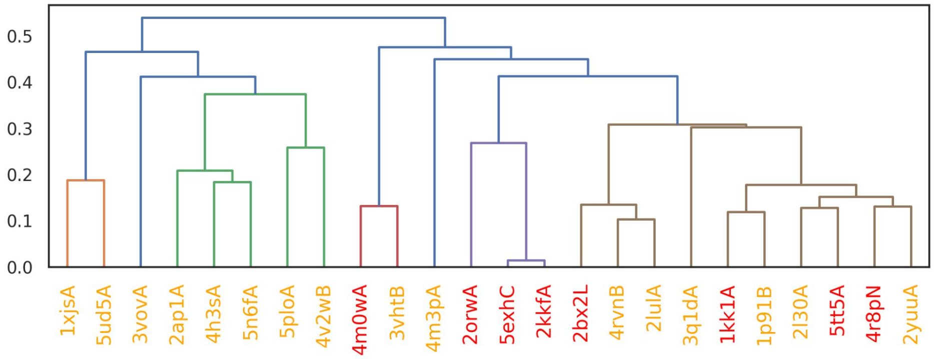

Figure S7: Hierarchical clustering results for sprite embeddings computed from high-precision test-set examples. X-axis labels are colored by the number of cysteine residues coordinating the central zinc ion, which is a key feature that is correlated with the resulting clusters: orange=3, red=4.
